# Prevalence of glucose-6-phosphate dehydrogenase deficiency (G6PDd) and clinical implication for safe use of primaquine in malaria-endemic areas of Hainan Province, China

**DOI:** 10.3389/fpubh.2022.1010172

**Published:** 2022-10-21

**Authors:** Wen Zeng, Ning Liu, Yuchun Li, Ai Gao, Mengyi Yuan, Rui Ma, Na Jiang, Dingwei Sun, Guangze Wang, Xinyu Feng

**Affiliations:** ^1^Department of Tropic Disease, Hainan Center for Disease Control and Prevention, Haikou, China; ^2^Department of Gastrointestinal Surgery, Hainan General Hospital, Hainan Affiliated Hospital of Hainan Medical University, Haikou, China; ^3^Department of Biology, College of Life Sciences, Inner Mongolia University, Hohhot, China; ^4^National Institute of Parasitic Diseases, Chinese Center for Disease Control and Prevention (Chinese Center for Tropical Diseases Research), NHC Key Laboratory of Parasite and Vector Biology, WHO Collaborating Centre for Tropical Diseases, National Center for International Research on Tropical Diseases, Joint Research Laboratory of Genetics and Ecology on Parasite-Host Interaction, Fudan University, Shanghai, China; ^5^School of Global Health, Chinese Center for Tropical Diseases Research, Shanghai Jiao Tong University School of Medicine, One Health Center, Shanghai Jiao Tong University-The University of Edinburgh, Shanghai, China

**Keywords:** G6PD, primaquine, *vivax* malaria, spatial cluster, prevalence

## Abstract

Primaquine, the only licensed antimalarial drug for eradication of *Plasmodium vivax* and *Plasmodium ovale* malaria, may cause acute hemolytic anemia in individuals with glucose-6-phosphate dehydrogenase deficiency (G6PDd) during treatment. The different prevalence and distribution patterns of G6PDd in Hainan, the ancient malaria-endemic area, are unclear. This study included 5,622 suspected malaria patients between 2009 and 2011 in 11 counties of Hainan. Glucose-6-phosphate dehydrogenase deficiency prevalence was determined using the fluorescent spot test (FST) and malaria patients was confirmed by a positive light microscopy. The G6PDd prevalence for different ethnic groups, genders, and counties were calculated and compared using χ^2^-test. Spatial cluster and Spearman rank correlation of G6PDd prevalence and malaria incidence were analyzed. The overall G6PDd prevalence of study population was 7.45%. The G6PDd prevalence of males, Li ethnic minority, and malaria patients was significantly higher than that of females, Han ethnic majority, and non-malarial patients (*p* < 0.01), respectively. The spatial cluster of G6PDd and malaria located in south-western and central-southern Hainan, respectively, with no significant correlation. The study provides essential information on G6PDd prevalence in ancient malaria-endemic areas of Hainan Province. We also highlight the need for a better understanding of the mechanisms underlying the relationship between G6PDd prevalence and malaria incidence. These findings provide a reference for the safety of the primaquine-based intervention, even after malaria elimination.

## Introduction

The malaria elimination program started in 2010 (1) and succeeded in 2021 in China. Throughout the journey, Hainan Province, the most southern province of China and the most seriously affected malaria-endemic area was always the hot spot for the campaign. Since 2010, no falciparum malaria cases have been reported in the area. However, *Plasmodium vivax* malaria sustained endemic for another 2 years, presenting a critical barrier to elimination in Hainan Province and other countries planning to eliminate malaria. Notably, *P. vivax* malaria often occurs with low parasitemia and can be missed under routine surveillance. Furthermore, it has gradually become a consensus that *P. vivax* malaria can be as debilitating as falciparum malaria. Recently, imported *P. vivax* malaria has been documented and accounted for more than 80% of reported malaria cases. Therefore, additional measures are still needed to extend the comprehensive response to *P. vivax* malaria control by reducing *P. vivax* transmission/relapse, and meanwhile, protecting the most vulnerable populations.

There is increasing use of chemopreventive agents for the eradication of malaria toward malaria-free status. In China, primaquine is the only drug approved by the State Food and Drug Administration (SFDA) of China to prevent malaria relapse. It is one of the critical components of the nationwide malaria elimination program (2). Therefore, a massive primaquine administration was expected and applied in malaria-endemic regions. However, primaquine may cause acute hemolytic anemia in individuals with glucose-6-phosphate dehydrogenase deficiency (G6PDd), a common congenital X-linked hereditary enzyme deficiency widespread across most malaria-endemic countries (3). In order to improve the compliance rate of primaquine for the radical cure of malaria, it is urgent to understand better the distribution pattern of G6PDd in main malaria-endemic areas in China.

Previous studies have revealed a relatively low incidence of G6PDd, about 3–7% in China (3); however, there may still be a large number of people with G6PDd in the country, considering its large population. In addition, G6PDd prevalence varied significantly among ethnic groups (4–6). For example, the G6PDd prevalence of the ethnic minorities is different from that of the Han ethnic majority in Yunnan Province, one of the ancient malaria-endemic areas in China (7, 8). Hainan Province still has a multi-ethnic population, but the G6PDd prevalence of its various ethnic minorities remains unclear.

Generally speaking, G6PDd confers a protective effect against both falciparum malaria and vivax malaria episodes (9), lending to a presumptive consensus that wide geographical distribution of G6PDd in human populations is derived by the epidemic (9). Interestingly, there is ecological overlap between G6PDd and malaria endemic areas, as shown in Africa, southern Europe, the middle east, southeast Asia, and the central and southern Pacific islands (10, 11). Therefore, information on the prevalence of G6PDd is critical to optimize the malaria elimination strategy, especially for radical treatment of *P. vivax* malaria using primaquine. It is necessary to determine the G6PDd distribution patterns in malaria-endemic areas and further analyze the unveiled relationship between malaria incidence and the G6PDd prevalence.

The present study presented the most recently updated information on G6PDd in Hainan, China. We also analyzed and compared the G6PDd distribution patterns among different counties, ethnic groups, and genders and explored the relationship between the spatial distribution of malaria cases and G6PDd status in the minority area. These results would be beneficial for optimizing existing tools against *P. vivax*, and deploying more effective measures for protecting the most vulnerable populations.

## Materials and methods

### Study area

The study areas were located on the main island of Hainan Province, 18°10′-20°10′ north latitude and 108°37′-111°03′ east longitude, in southern China. This region has a land area of 33,900 km^2^ with a population of 8.26 million. There are multi-ethnic populations, including Li, Miao, and Hui ethnic minorities living together in the central and southern mountainous areas. The Han ethnic majority distributes throughout the island. The Li ethnic minority is one of the earliest inhabitants and the most populous ethnic minority in Hainan.

### Ethics statement

This clinical study protocol was reviewed and approved by the Ethics Review Committee of the Hainan Center for Disease Control and Prevention. Each participant provided informed consent (in Chinese) before participating in this study. In most cases, the participants provided their written informed consent. The consent was verbal for patients who could not read or write standard Chinese. In these cases, the research nurse documented the participant's consent in writing, including the contents and methods of information provided to the participant and the date and time of the verbal consent, which was then witnessed and signed by another nurse who was not in the research team. The informed consent record, either written or verbal, was kept in the participant's hospital chart. The Ethics mentioned above Committee reviewed and approved this consent procedure.

### Participants and data collection

We selected 11 malaria-endemic counties (Baisha, Baoting, Changjiang, Dongfang, Ledong, Lingshui, Qionghai, Qiongzhong, Sanya, Wanning, and Wuzhishan) in Hainan according to the reported malaria incidence in the previous 5 years (2004–2009). We selected one comprehensive county-level hospital in each county as the investigation sites. Patients with malaria-like symptoms such as fever, shivering, and perspiring seeking medical service in these 11 hospitals between January 1, 2009 and December 31, 2011 were enrolled. Each participant's demographic and clinical information was collected, including the ethnic group, sex, age, location of residence, clinical symptom, and the routine test.

### Procedure for detection of G6PDd

The peripheral blood sample (5 ml) was obtained from each participant by forearm venipuncture and tested for G6PDd using the fluorescent spot test (FST) method as previously described (12, 13). Briefly, 10 μl of blood sample was added to 100 μl test reagents and incubated at 37°C for 30 min; a spot was made on ordinary filter paper and was permitted to dry; the spots were then visualized under an ultraviolet (UV) light. Spots that showed fluorescence were classified as normal G6PD, and spots that failed to show fluorescence were classified as G6PDd. After being used for G6PDd test, microscopy test, and blood routine test, the remaining blood samples were stored in Hainan CDC for further confirmation assays. The microscopy examined all patients to confirm or rule out a malaria diagnosis.

### G6PDd prevalence and spatial distribution analysis

The G6PDd prevalence for various groups, including different ethnic groups, genders, counties, malaria patients, and patients without malaria, were calculated and compared using the χ^2^-test. In order to avoid the confounding effects caused by ethnicity in different counties, we calculated the ethnic-standardized G6PD prevalence. The ethnic-standardized G6PDd prevalence of the study area and each county were calculated and compared with their corresponding general G6PDd prevalence using the χ^2^-test. The malaria incidence of the study participants in each county were calculated.

The G6PDd prevalence of each county were first categorized into three levels, i.e., 0–5.00%, 5.01–10.00%, and 10.01–15.00%, respectively, and then geo-coded and matched to the corresponding polygon on a digital map of Hainan, which was marked with different colors to represent the different levels of the G6PDd prevalence. The spatial autocorrelations of the G6PDd prevalence and ethnic-standardized G6PDd prevalence across the study area was estimated using Moran's I statistic program to determine whether G6PDd was randomly distributed among the counties. The spatial analyses were conducted using the Spatial Analyst Model with ArcGIS 9.2 software (ArcGIS 9.2, Environmental Systems Research Institute, Redlands, CA, USA).

### Cluster analysis

The spatial cluster analyses of malaria incidence and G6PDd prevalence were conducted between 2009 and 2011. In order to detect and compare the counties with a high risk of malaria and G6PDd at different spatial scales, maximum spatial cluster sizes of 20%, 30%, and 40% of the entire population were specified for both malaria incidence and G6PDd prevalence. The cluster window with the highest likelihood ratio (LLR) was the most likely cluster to have the highest risk of malaria or G6PDd. The cluster window with the next to maximum LLR was the secondary likely cluster with the second-highest risk of malaria or G6PDd. The relative risks of malaria or G6PDd within and outside these windows were calculated to evaluate the degree of risk. The cluster analysis was performed using SatScan 7.0.3 (SatScan 7.0.3, Information Management Services Inc., Boston, MA, USA). Clustering was performed using purely spatial and a Poisson model was used during the analysis.

### Correlation analysis

Spearman rank correlation analysis was conducted to detect the relationship between G6PDd prevalence and malaria incidence in the study areas. The index *r* > 0 denoted a positive correlation, while *r* < 0 denoted a negative correlation. The correlation was considered significant when *P* < 0.05.

## Results

### Characteristics of the participants

Among the 5,622 participants in the present study, 3,444 were Han Chinese and 2,178 were Li Chinese, including 3,009 males and 2,613 females, and 670 were malaria patients (including 667 *P. vivax malaria* patients and 3 *Plasmodium falciparum* malaria patients) and 4,952 were patients without malaria (Supplementary Table S1). The age of the 5,622 participants ranged from 19 to 72 years old (median = 39 years, IQR 26–52 years). There were 634 (94.63%) malaria patients with a body temperature higher than 37.5°C, 571 (85.22%) with shivering, 595 (88.81%) with perspire, 206 (30.75%) with abdominal pain, 113 (16.87%) with nausea, and 105 (15.67%) with vomiting. The median WBC, RBC, and hemoglobin of the 670 malaria patients were 8.2 × 10^9^/L (4.6–11.3 × 10^9^/L), 4.3 × 10^12^/L (3.5–5.2 × 10^12^/L), and 121 g/L (102–139 g/L).

### G6PDd prevalence

The overall G6PDd prevalence was 7.45% (419/5,622). The G6PDd prevalence of males was significantly higher than that of females (8.91% vs. 5.78%; χ^2^ = 19.84, *P* < 0.01, Table 1). The G6PDd prevalence of the Li ethnic minority was significantly higher than that of the Han ethnic majority (12.03% vs. 4.56%; χ^2^ = 107.96, *P* < 0.01, Table 1). The G6PDd prevalence of malaria patients was significantly higher than that of patients without malaria (12.39% vs. 6.79%; χ^2^ = 26.86, *P* < 0.01, Table 1). The G6PDd prevalence of male patients was significantly higher than that of patients without malaria (12.88% vs. 8.36%; χ^2^ = 8.07, *P* < 0.01). After a careful comparative analysis of the subgroups, Li ethnic group, male gender, and G6PDd were at risk of malaria. There was no significant difference between the G6PDd prevalence and the ethnic-standardized G6PDd prevalence of the entire study population (7.45% vs. 8.29%; χ^2^ = 3.55, *P* > 0.05).

**Table 1:** The G6PDd prevalence in different groups in Hainan, China, 2009–2011.

**Variable**	**Subgroup (*N*)**	**G6PDd (%)**	**Prevalence (%)**	**χ^2^**	***P***
Ethnicity	Li (*N* = 3,009)	268	8.91	19.84	<0.01
	Han (*N* = 2,613)	151	5.87		
Gender	Male (*N* = 2,178)	262	12.03	107.96	<0.01
	Female (*N* = 3,444)	157	4.65		
Malaria status	Malaria (*N* = 670)	83	12.39	26.86	<0.01
	Non-malaria (*N* = 4,952)	336	6.79		

### Spatial distribution pattern of G6PDd

**Figure 1:**
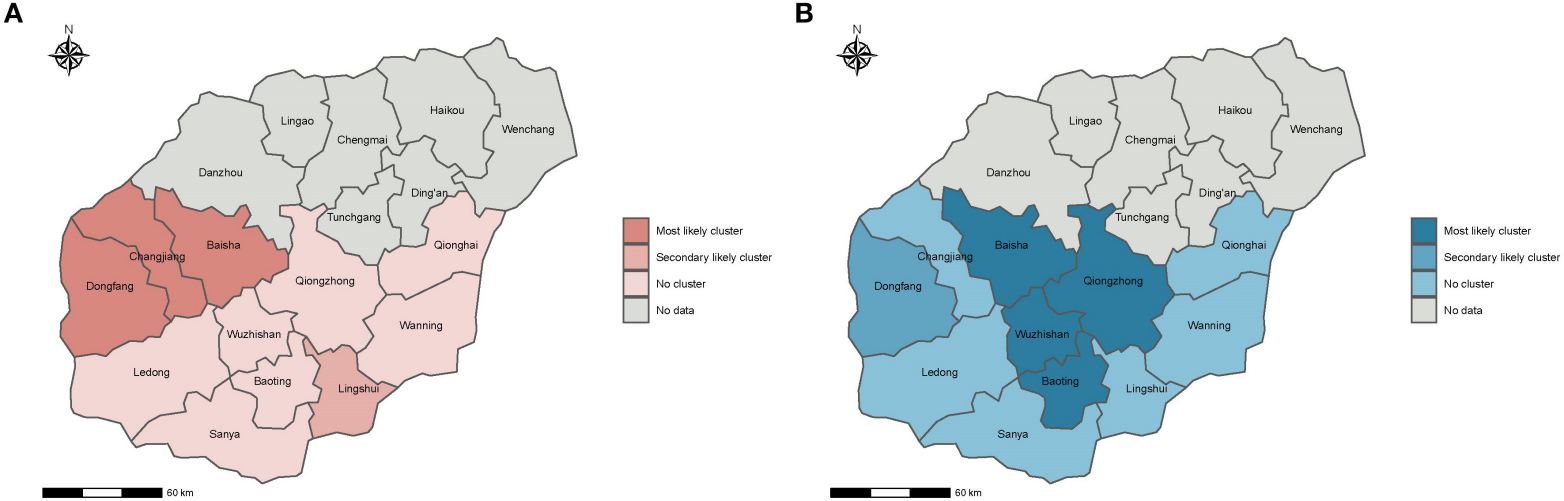
Spatial distribution of clusters with high G6PDd risk and high malaria risk in Hainan, China, 2009–2011. **(A,B)** is the spatial distribution of clusters with high G6PDd risk and high malaria risk, respectively. The risk of G6PDd and malaria were ordered using the likelihood ratio (LLR). The most likely cluster indicates the area with the highest risk of G6PDd and malaria, and the secondary likely cluster indicates the area with the second highest risk of G6PDd and malaria.

The G6PDd prevalence varied from 2.96% in Qionghai County to 14.48% in Dongfang County, and the ethnic-standardized G6PDd prevalence varied from 2.11% in Wuzhishan County to 14.82% in Dongfang County (Table 2). There were significant differences in both G6PDd prevalence and ethnic-standardized G6PDd prevalence among the 11 counties (Table 2, χ^2^ = 121.16, *P* < 0.01; χ^2^ = 110.65, *P* < 0.01). In addition, significantly positive spatial autocorrelations of G6PDd prevalence and ethnic-standardized G6PDd prevalence across the 11 counties [Moran's I = 0.49, Z (I) = 2.62, *P* < 0.05; Moran's I = 0.53, Z (I) = 2.71, *P* < 0.05] was observed. However, there was no significant difference between the G6PDd prevalence and the ethnic-standardized G6PDd prevalence in each county. The spatial distribution pattern of the G6PDd prevalence of the 11 counties was heterogeneous (Figure 1).

**Table 2:** The G6PDd prevalence, ethnic-adjusted G6PDd prevalence, and malaria incidence in 11 counties in Hainan, China, 2009–2011.

**County**	**Number of study participants**	**Number of malaria cases**	**Malaria incidence (%)**	**Number of G6PDd cases**	**G6PDd prevalence (%)**	**χ^2^**	***P***	**Ethnic-adjusted G6PDd prevalence (%)**	**χ^2^**	***P***	χ^2*^	P*
Baisha	423	102	24.11	60	14.18	121.16	<0.01	11.33	110.65	<0.01	1.53	0.22
Baoting	469	126	26.87	29	6.18			5.03			0.50	0.48
Changjiang	566	58	10.25	43	7.60			8.94			0.74	0.39
Dongfang	504	70	13.89	73	14.48			14.82			0.03	0.86
Ledong	485	42	8.66	22	4.54			4.97			0.09	0.76
Lingshui	388	59	15.21	49	12.63			10.56			0.80	0.37
Qionghai	608	36	5.92	18	2.96			4.88			3.12	0.08
Qiongzhong	460	79	17.17	29	6.30			5.17			0.50	0.48
Sanya	489	20	4.09	40	8.18			8.55			0.05	0.82
Wanning	450	42	9.33	25	5.56			7.19			0.92	0.34
Wuzhishan	780	36	4.62	31	3.97			2.11			4.94	0.03

*χ^2^-test of the difference between the G6PDd prevalence and the ethnic-adjusted G6PDd prevalence in each county.

### Spatial clusters of G6PDd prevalence and malaria incidence

We limit the maximum spatial cluster size to 20% of the population size at risk, three counties constituted the most likely cluster of G6PDd (Baisha Changjiang and Dongfang) and one county constituted the secondary likely cluster (Lingshui) (Figure 1A). When the maximum spatial cluster size was 20% of the population size, four counties constituted the most likely cluster of malaria (Lingshui, Qiongzhong, Wuzhishan, and Baoting), and one county constituted the secondary likely cluster (Baisha) (Figure 1B). These results further indicated that the distribution patterns of G6PDd and malaria were significantly different among the studied counties.

### Correlation between G6PDd prevalence and malaria incidence

Correlation analysis indicated no significant correlation between the G6PDd prevalence and malaria incidence during 2009–2011 in the study areas (*r* = 0.46, *P* > 0.05).

## Discussion

It has been estimated that more than 400 million people are affected by G6PDd in malaria-endemic regions worldwide (10). Moreover, G6PDd prevalence in the ethnic minority areas is most likely underestimated due to no universal access to diagnosis or high expenditure (7, 14–18). So, the actual G6PDd prevalence may need continuous updates leading to additional difficulty in combating malaria (3, 19). The present study demonstrated that the population in Hainan has a significantly higher G6PDd prevalence compared with other areas in China (8, 9, 20).

Though currently, Hainan has eliminated *P. vivax* malaria, the current undated data on G6PDd prevalence has important implications for treating imported *P. vivax* malaria and preventing re-transmission in the future.

Glucose-6-phosphate dehydrogenase (G6PD) deficiency is a common X-linked genetic trait, and thus affects mainly males. Link between G6PDd and gender revealed that the prevalence in males is usually higher than that of females. The G6PD test classifies the heterozygous females as normal because they have both normal and deficient G6PD red blood cells. From the genetic point of view, our result confirmed that the G6PDd prevalence of males is significantly higher than that of females overall (12.03% vs. 4.56%) or at the group level (both in Li ethnic group and Han ethnic group). On the other hand, males are more likely to become infected with malaria because of some social and behavioral characteristics. For example, males are more likely exposed to the environment of Anopheles mosquitoes, and females are more likely to use mosquito nets than males as they tend to protect their young children. Where feasible, particular attention should be paid to the males, especially in the endemic area or where an outbreak happens when massive primaquine is used.

Knowing the G6PDd prevalence is crucial to determining whether primaquine is contraindicated in patients (21, 22). Hainan Province is multi-ethnic with variant genetic backgrounds, while Han and Li are the two main ethnic groups. According to a national survey, the G6PDd prevalence among ethnic groups is usually greater than among ethnic Han Chinese. In the present study, we also found the different G6PDd prevalence among ethnic groups. For example, the G6PDd prevalence (8.91%) of the Li ethnic minority was significantly higher than that of the Han ethnic majority and was also higher than that of other ethnic minority populations reported from other areas in China (8, 20, 23). Li ethnic minority group was the second largest population with unique genetic traits on Hainan Island. However, the G6PDd studies on Hainan Li population are still insufficient because Li populations live in remote or hard-to-reach areas and have limited access to health facilities. Particular attention should be paid to high-risk groups vulnerable to drug reactions, especially when their genetic variants are unknown.

Glucose-6-phosphate dehydrogenase deficiency can provide protection from severe malaria (24). There is significant selective pressure on the *G6PD* gene in malaria-endemic areas resulting G6PDd prevalence is exceptionally high in some areas of Africa, the Middle East, and South Asia (25). The present study also found that the G6PDd prevalence in malaria patients was more remarkable when compared with that in the non-malaria patient. However, its protective effect on preventing severe malaria needs further evaluation because we did not find such patients in our investigation period. Additionally, the relationship between parasite density and G6PDd status has yet to be explored.

We also found that the distribution patterns of G6PDd were not consistent with the malaria incidence by cluster analysis. These results suggest that the spatial correlation between G6PDd prevalence and malaria incidence is not evident in our study. Similarly, Rosalind et al. established a geostatistical model based on G6PDd prevalence, estimated affected populations, and found that G6PDd was spatially heterogeneous. However, cluster analysis revealed that malaria patients were mainly distributed in the central-southern counties of Hainan, consistent with the epidemic situation during the study period (26). Identification of distribution pattern of G6PD and malaria incidence can provide valuable insights for deliberating the relatedness.

Of note, the present study also has some limitations. Firstly, we only included a small number of participants from 11 counties, which may not reflect the true representative of the overall demographic sample. Secondly, we only focused on the two largest ethnic groups but not include other ethnic minorities. The G6PDd patterns and factors influencing the associations with malaria incidence are needed to examine in a stratified sampling method. Thirdly, the enrolled participants were selected from the hospital instead of the community, which may lead to bias.

## Conclusion

In summary, the results of this study represent the actual G6PDd status in malaria-endemic areas of Hainan. The Li ethnic minority had a higher G6PDd prevalence, especially in males and malaria patients. The G6PDd prevalence was not spatially correlated with the malaria incidence. Thus, in order to mitigate the risk of primaquine-induced hemolysis, special attention should be paid to minority populations in Hainan. More efficient and convenient diagnostic testing tools, such as point-of-care field tests, are expected to inform the potential risk of primaquine-associated harm.

## Data availability statement

The original contributions presented in the study are included in the article/Supplementary material, further inquiries can be directed to the corresponding authors.

## Ethics statement

This clinical study protocol was reviewed and approved by the Ethics Review Committee of the Hainan Center for Disease Control and Prevention. Each participant provided informed consent (in Chinese) before participating in this study. In most cases, the participants provided their written informed consent. The consent was verbal for patients who could not read or write standard Chinese. In these cases, the research nurse documented the participant's consent in writing, including the contents and methods of information provided to the participant and the date and time of the verbal consent, which was then witnessed and signed by another nurse who was not in the research team. The informed consent record, either written or verbal, was kept in the participant's hospital chart. The Ethics mentioned above Committee reviewed and approved this consent procedure. The patients/participants provided their written informed consent to participate in this study.

## Author contributions

WZ, YL, and GW contributed to the original idea and conceived the paper. GW, NL, AG, MY, RM, and NJ wrote the initial draft of the paper. XF, DS, and GW contributed to the revision of the manuscript. XF reviewed the final version. All authors approved the final manuscript.

## Funding

This study was supported by the Key Medical Research Project of the Health Department of Hainan Province (2010Qiong-36) and the Hainan Provincial Scientific Research Grant (813251). This study was also supported by Hainan Provincial Basic and Applied Basic Research Program (Natural Science Foundation) for High-level Talents in 2019 (2019RC394).

## Conflict of interest

The authors declare that the research was conducted in the absence of any commercial or financial relationships that could be construed as a potential conflict of interest.

## Publisher's note

All claims expressed in this article are solely those of the authors and do not necessarily represent those of their affiliated organizations, or those of the publisher, the editors and the reviewers. Any product that may be evaluated in this article, or claim that may be made by its manufacturer, is not guaranteed or endorsed by the publisher.
